# Current Strategies and Novel Therapeutic Approaches for Metastatic Urothelial Carcinoma

**DOI:** 10.3390/cancers12061449

**Published:** 2020-06-02

**Authors:** Veronica Mollica, Alessandro Rizzo, Rodolfo Montironi, Liang Cheng, Francesca Giunchi, Riccardo Schiavina, Matteo Santoni, Michelangelo Fiorentino, Antonio Lopez-Beltran, Eugenio Brunocilla, Giovanni Brandi, Francesco Massari

**Affiliations:** 1 Division of Oncology, S.Orsola-Malpighi Hospital, 40138 Bologna, Italy; veronica.mollica7@gmail.com (V.M.); rizzo.alessandro179@gmail.com (A.R.); giovanni.brandi@unibo.it (G.B.); 2 Department of Experimental, Diagnostic and Specialty Medicine, S.Orsola-Malpighi Hospital, 40138 Bologna, Italy; 3 Section of Pathological Anatomy, Polytechnic University of the Marche Region, School of Medicine, United Hospitals, 60121 Ancona, Italy; r.montironi@staff.univpm.it; 4 Department of Pathology and Laboratory Medicine, Indiana University School of Medicine, Indianapolis, IN 46202, USA; liang_cheng@yahoo.com; 5 Pathology Service, Addarii Institute of Oncology, S-Orsola-Malpighi Hospital, 40138 Bologna, Italy; frachikka@virgilio.it; 6 Department of Urology, University of Bologna, S-Orsola-Malpighi Hospital, 40138 Bologna, Italy; riccardo.schiavina3@unibo.it (R.S.); eugenio.brunocilla@unibo.it (E.B.); 7 Oncology Unit, Macerata Hospital, 62100 Macerata, Italy; mattymo@alice.it; 8 Department of Pathology, University of Bologna, School of Medicine, 40126 Bologna, Italy; michelangelo.fiorentino@unibo.it; 9 Unit of Anatomical Pathology, Faculty of Medicine, Cordoba University, 14071 Cordoba, Spain; em1lobea@gmail.com

**Keywords:** urothelial carcinoma, immunotherapy, immune checkpoint inhibitors, FGFR, antibody drug conjugates, clinical trials, PD-1, PD-L1

## Abstract

Urothelial carcinoma (UC) is a frequent cause of cancer-related deaths worldwide. Metastatic UC has been historically associated with poor prognosis, with a median overall survival of approximately 15 months and a 5-year survival rate of 18%. Although platinum-based chemotherapy remains the mainstay of medical treatment for patients with metastatic UC, chemotherapy clinical trials produced modest benefit with short-lived, disappointing responses. In recent years, the better understanding of the role of immune system in cancer control has led to the development and approval of several immunotherapeutic approaches in UC therapy, where immune checkpoint inhibitors have been revolutionizing the treatment of metastatic UC. Because of a better tumor molecular profiling, FGFR inhibitors, PARP inhibitors, anti-HER2 agents, and antibody drug conjugates targeting Nectin-4 are also emerging as new therapeutic options. Moreover, a wide number of trials is ongoing with the aim to evaluate several other alterations and pathways as new potential targets in metastatic UC. In this review, we will discuss the recent advances and highlight future directions of the medical treatment of UC, with a particular focus on recently published data and ongoing active and recruiting trials.

## 1. Introduction

Urothelial carcinoma (UC) is a common cancer worldwide, with nearly half a million of new diagnoses annually [1]. Although UC includes a group of tumors of the bladder, renal pelvis, ureter and urethra, more than 90% of UCs occurs in the lower urinary tract, therefore involving urinary bladder and, less often, urethra [2]. About 70–75% of patients at diagnosis are affected by non-muscle-invasive bladder cancer (NMIBC) while more than 25% of cases are already muscle-invasive bladder cancer (MIBC) or metastatic forms [3]. Despite recent improvements in the field of medical oncology, the prognosis of patients with advanced or metastatic UC remains dismal, with a median overall survival (OS) of approximately 15 months from diagnosis [4]. In the last twenty years, front-line cisplatin-based chemotherapy represented the mainstay of palliative treatment for UC, with combinations such as gemcitabine plus cisplatin (GC) and methotrexate, vinblastine, doxorubicin, and cisplatin (M-VAC) as the cornerstones of standard treatment in advanced or metastatic UC [5,6,7]. Although GC and M-VAC regimens showed similar outcomes in terms of OS and time to treatment failure (TTF), GC is commonly preferred over M-VAC on the basis of lower mucosal and hematological toxicity [5,6,7]. Nevertheless, most patients are cisplatin-ineligible because of inadequate renal function, poor Eastern Cooperative Oncology Group (ECOG) performance status, peripheral neuropathy, old age and/or other underlying comorbidities, and thus, cisplatin is usually replaced by carboplatin in unfit patients, as we shall see later [8]. Unfortunately, after the failure of first-line treatments, further therapies have yielded poor response rates and the overall results obtained with conventional cytotoxic agents (as monotherapy or in combination) have been far from being satisfactory since UC patients historically carried a median OS of approximately 12–17 months [9]. Thus, there is an urgent need for novel, more effective treatment options in advanced or metastatic UC.

Recent phase I to III studies with drugs targeting immune checkpoints and different molecular pathways of UC are ongoing and some were published in the last three years [10]. These novel agents primarily include immune checkpoint inhibitors (ICIs), tyrosine kinase inhibitors (TKIs) targeting fibroblast growth factor receptor (FGFR), and antibody-drug conjugates (ADCs) directed against Nectin-4 [11,12]; however, many other alterations and pathways are also emerging as new potential targets [13]. In this paper, we provide a comprehensive review of recent trials and the current state of ongoing active and recruiting Phase I, II, and III trials according to clinicaltrial.gov, looking into the future of the rapidly evolving landscape of medical treatment for advanced or metastatic UC.

We performed a research on Pubmed/Medline, Cochrane library and Scopus using the keyword “urothelial carcinoma” OR “bladder carcinoma” OR “bladder urothelial carcinoma” OR “bladder cancer” OR “bladder neoplasm.” We selected pivotal registration studies. We also selected the most relevant and pertinent studies considering quality of the studies in terms of their applicability, how they were conducted, statistical analysis, number of patients enrolled, outcomes. For ongoing clinical trials, we searched in the clinicaltrials.gov database for recruiting and active, not recruiting trials, using the following keywords: “urothelial carcinoma” OR “bladder carcinoma” OR “bladder urothelial carcinoma” OR “bladder cancer” OR “bladder neoplasm.” We restricted our research to phase 1, 2, or 3 trials focused on the metastatic/advanced setting.

## 2. Treatment Strategies: State-of-the-Art

### 2.1. Immune Checkpoint Inhibitors

**Figure 1:**
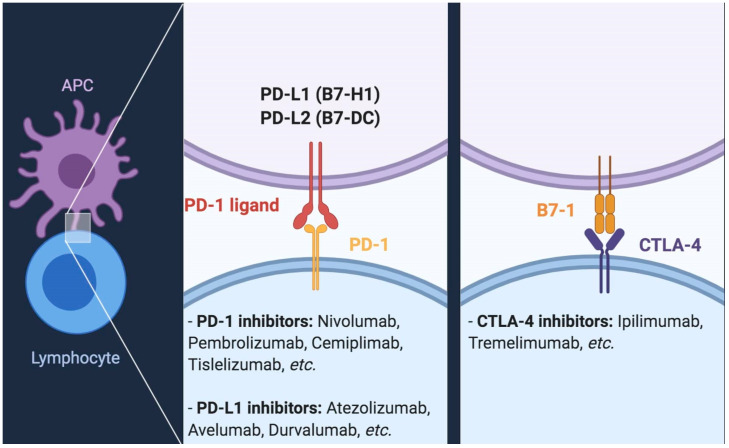
The interaction between PD-1/PD-L1 and CTLA-4/B7-1, a key mechanism exploited by immune checkpoint inhibitors. PD-1 inhibitors include nivolumab, pembrolizumab, cemiplimab, tislelizumab, and other agents currently in development; conversely, PD-L1 inhibitors encompass agents such as atezolizumab, avelumab, durvalumab, while CTLA-4 inhibitors encompass ipilimumab and tremelimumab.

The advent of ICIs blocking the interaction of Programmed Death 1 (PD-1) and Cytotoxic T-Lymphocyte Antigen 4 (CTLA-4) with their specific ligands has recently revolutionized the treatment of several hematological and solid malignancies (Figure 1) [14,15,16]. Outstandingly, ICIs have challenged previous treatment paradigms of most solid tumors, including the therapeutic decision-making approach to advanced or metastatic UC, in the first-line setting for cisplatin-ineligible patients as well as in the post-platinum setting [17,18]. Given the well-known activity of topical instillation of Bacillus of Calmette-Guérin (BCG) in high-risk, non-muscle invasive disease, UC immediately appeared as a suitable candidate for modern immunotherapy [2]; moreover, UC is known to be a highly antigenic malignancy, given the high rates of DNA alterations and mutations leading to the formation of neoantigens, an element which further supports the application of ICIs in advanced or metastatic UC [19,20,21]. In light of data provided by a variety of recent trials, the therapeutic scenario of UC is rapidly changing but, unfortunately, several unmet clinical needs still persist [21,22].

Although cisplatin-based regimens are considered the standard first-line treatment in advanced or metastatic UC, more than 50% of patients are ineligible for cisplatin in clinical practice [23]. For this non-negligible group of patients, carboplatin plus gemcitabine has been considered the standard treatment based on the results of the EORTC 30,986 trial, with several other combinations and agents showing less favorable safety profiles and inferior outcomes compared to cisplatin-based first-line therapy [24,25,26]. Thus, the modest survival benefits observed with available treatment options highlighted the need for new effective strategies [27] and for this purpose, following small phase I trials, the role of ICIs as front-line treatment in cisplatin-ineligible patients was investigated in KEYNOTE-052 and IMvigor210 trials [28,29]. 

The KEYNOTE-052 [28] was a phase II trial aimed to evaluate the safety and efficacy of pembrolizumab monotherapy (200 mg flat dose every three weeks) in 370 chemo-naive, cisplatin-ineligible patients. In this setting pembrolizumab, a highly selective humanized monoclonal IgG4 isotype antibody against PD-1 protein, produced an overall response rate (ORR) of 24% with 5% of complete response (CR). Interestingly, the magnitude of ORR and survival benefit was related to programmed death ligand-1 (PD-L1) expression: in fact, in patients with PD-L1 expression combined positive score (CPS) ≥ 10%, pembrolizumab resulted in improved survival, with a median OS of 18.5 months versus 11.5 months in overall cohort. Finally, the CPS ≥ 10% population reported higher ORR (37%) compared to the CPS < 10% subgroup of patients (ORR = 18%). 

The IMvigor210 trial [29] was a 2-cohort Phase 2 study; while cohort 2 assessed atezolizumab in a post-platinum setting, in cohort 1 the anti-PD-L1 agent was tested as first-line treatment in cisplatin-ineligible subjects. Total of 119 untreated patients were included in cohort 1 and received atezolizumab, 1200 mg flat dose every three weeks, achieving an ORR of 23% with CR and partial response (PR) of 9% and 12% respectively, regardless of PD-L1 expression. Clinical activity of atezolizumab was higher than those observed with systemic chemotherapies traditionally used in this setting, in respect of whom the anti-PD-L1 agent showed also a more manageable safety profile. 

Based on the aforementioned studies, pembrolizumab and atezolizumab were approved by the US Food and Drug Administration (FDA) and European Medicines Agency (EMA) for front-line use in cisplatin unfit patients affected by advanced or metastatic UC. However, the use of pembrolizumab and atezolizumab has been subsequently restricted, following early data from KEYNOTE-361 and IMvigor130 phase III trials which, as we shall explain later, are currently investigating combination chemo-immunotherapy in advanced or metastatic UC. In these two trials, patients with low expression of PD-L1 receiving single-agent ICI experienced worse survival compared to patients receiving standard chemotherapy [30,31].

Following platinum-based chemotherapy, large proportions of patients are either non-responders or relapsed, and therefore proceed for second-line treatment [32]. Until some years ago, taxanes or vinflunine were considered standard second-line treatments, despite disappointing ORRs and an overall modest clinical benefit [33,34]. In this scenario, recent results of Phase I to III studies with agents targeting PD-1 and PD-L1 have led to fast approval of ICIs as second-line treatments. In particular, five ICIs (two anti-PD-1 agents—pembrolizumab and nivolumab—and three anti-PD-L1 agents—atezolizumab, durvalumab, and avelumab) have been granted approval by FDA for patients with advanced or metastatic UC whose disease progressed during or following platinum-based chemotherapy [35]. Conversely, despite FDA has granted approval for the aforementioned agents, pembrolizumab is the only ICI that showed a survival benefit in a phase III randomized clinical trial and whose activity is supported by higher levels of evidence [36].

The approval of pembrolizumab in post-platinum setting was granted based on the results of the KEYNOTE-045 trial [37]. This phase III, open-label, randomized trial compared pembrolizumab (flat dose of 200 mg every three weeks) with chemotherapy by investigators’ choice, including vinflunine and taxanes, in patients who recurred or progressed after a platinum-based regimen. A higher ORR was observed in patients treated with pembrolizumab (21.1% vs. 11.4% of the chemotherapy arm); moreover, an OS benefit was observed, regardless of PD-L1 expression (in the overall population 10.3 and 7.4 months, in the immunotherapy and chemotherapy arm, respectively, hazard ratio (HR) 0.73; 95% confidence interval (CI) 0.59 to 0.91; *p* = 0.002). Finally, pembrolizumab was associated with fewer grade 3–4 adverse events compared to vinflunine, paclitaxel, and docetaxel. 

Instead, the activity of atezolizumab was tested in the phase II IMvigor210 and the phase III IMvigor211 trials [38,39]. As stated above, the IMvigor210 trial was a 2-cohort phase II study aimed to evaluate the efficacy and safety of atezolizumab (1200 mg flat dose every three weeks) in untreated, cisplatin-ineligible patients (cohort 1) as well as in patients whose disease was refractory to platinum-based chemotherapy (cohort 2) [38]. In the cohort 2, including 315 eligible subjects, an ORR of 15% was observed, with a sustained response duration and an acceptable safety profile; moreover, in patients presenting PD-L1 expression ≥5% the ORR was higher (27%) and the survival benefit longer compared to the PD-L1 ≥ 1 and < 5% cohort and the PD-L1 < 1% group. On the basis of these promising findings, the role of atezolizumab was further assessed in the confirmatory phase III, open-label, randomized IMvigor211 trial [39], which compared atezolizumab to chemotherapy by investigators’ choice, including vinflunine and taxanes, in patients who recurred or progressed after a platinum-based regimen. The primary endpoint, OS in patients with PD-L1 expression ≥ 5%, did not significantly differ between the two arms, with a median OS of 11.1 and 10.6 months in atezolizumab and chemotherapy arms, respectively (HR 0.87; 95% CI 0.63–1.21; *p* = 0.41). Despite the negative primary endpoint, IMvigor211 provided useful data in terms of median duration of response, which was significantly higher in the ICI arm (15.9 vs. 8.3 months; HR 0.57; 95% CI 0.26–1.26) and in terms of toxicity, with the PD-L1 inhibitor confirming a manageable safety profile. Finally, the exploratory analysis of the intention to treat population showed a survival benefit for atezolizumab (HR 0.85; 95% CI 0.73–0.99). 

Nivolumab is a human monoclonal IgG4 antibody that blocks the human PD-1 receptor, whose efficacy in the post-platinum setting was explored in the CheckMate 275 trial [40]; in this phase II trial, nivolumab (240 mg flat dose every two weeks) showed an ORR of 20% with 2% CR in 270 patients affected by advanced or metastatic UC. With regard to PD-L1 expression, ORR was significantly higher in the subgroup of patients with PD-L1 expression ≥5% (28.4%) compared to the PD-L1 ≥ 1% (23.8%) and the PD-L1 negative (16.1%) cohorts.

A similar level of activity was observed with post-platinum avelumab and durvalumab in the multicohort phase Ib JAVELIN trial [41] and the single-arm, phase I/II Study 1108 [42], respectively. Avelumab (10 mg/kg every two weeks), an anti-PD-L1 antibody that blocks the binding of PD-L1 to PD-1, reported an ORR of 17% with 6% CR in platinum-refractory or cisplatin unfit patients; interestingly, in PD-L1 negative subgroup ORR fell to 9% while reached the 40% in PD-L1 ≥ 5% patients. 

Similarly, considering the cutoff of 25% of PD-L1 expression (assessed with immunohistochemistry (IHC) on tumor tissue via Ventana SP263 assay) in Study 1108, the subgroup of patients with PD-L1^high^ achieved higher response rates and survival benefit compared to PD-L1^low^ cohort (20 vs. 8 months) with the anti-PD-L1 human IgG1 durvalumab [42]. In Study 1108, patients were administered durvalumab intravenous infusion, 10 mg/kg every 2 weeks. 

Additional data from a number of ongoing prospective clinical trials will help to confirm the activity of ICIs in previously treated and untreated patients [43]; in the era of precision, tailor-made oncology, several questions are still unanswered, including the identification of predictive biomarkers, sequential treatment strategies, and proper selection of patients in advanced or metastatic UC. A non-negligible unanswered question is how to assess PD-L1 expression. For example, in KEYNOTE-052 and IMvigor210 PD-L1 cutoff was different and it was assessed differently; in KEYNOTE-052 PD-L1 positive tumors were those presenting a CPS ≥ 10% and PD-L1 expression in formalin-fixed, paraffine-embedded tissue was determined using the PD-L1 clinical trial assay (PD-L1 IHC 22C3 pharmDx assay; Agilent Technologies, Carpinteria, CA, USA). Differently, in the IMvigor210 trial the VENTANA SP142 immunohistochemistry assay (Ventana Medical Systems, Inc.; Tucson, AZ, USA) was used to evaluate PD-L1 expression on tumor-infiltrating immune cells (IC) and a scoring criteria designated tumors as IC0, IC1, or IC2/3 (PD-L1 expression on <1%, ≥1% and <5%, or ≥5% of IC, respectively).

### 2.2. Target Therapies

**Figure 2:**
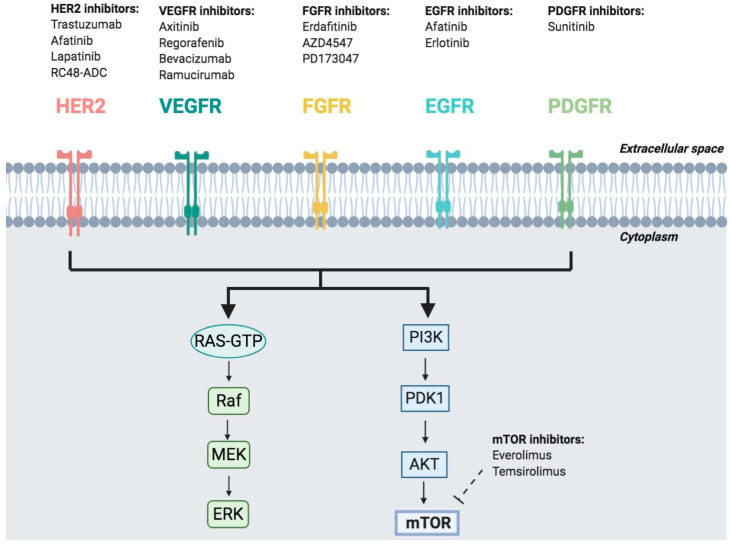
Frequent potentially actionable mutations and pathways involved in UC. EGFR: epidermal growth factor receptor; ERK: mitogen-activated protein kinase; FGFR: fibroblast growth factor receptor; HER2: receptor tyrosine-protein kinase ERBB2; MAPK: mitogen-activated protein kinase; MEK: dual-specificity mitogen-activated protein kinase; mTOR: mammalian target of rapamycin; PDGFR: platelet-derived growth factor receptor; PI3K: phosphoinositide 3-kinase; PTEN: phosphatase and tensin homologue; VEGFR: vascular endothelial growth factor receptor.

In the recent years, genomic characterization of advanced-stage UC has given an insight on which are molecular drivers at the basis of the oncogenesis and progression of UC and that could be potentially targetable (Figure 2) [44]. The Cancer Genome Atlas (TCGA) project for bladder cancer had the purpose to provide a comprehensive landscape of molecular alterations [45]. The first integrated analysis on 131 UC demonstrated statistically significant recurrent mutations in 32 genes. Furthermore, this analysis showed that 69% of the tumors presented potential therapeutic targets, of which 42% regarded the phosphatidylinositol-3-kinase (PI3K)/AKT/mammalian target of rapamycin (mTOR) pathway and 44% in the receptor tyrosine kinase/MAPK pathway, and identified an in-frame activating *FGFR3-TACC3* fusion in three tumors [45]. Alterations in the PI3K/AKT/mTOR pathway consisted in point mutations in *PIK3CA* (17%), mutation or deletion of *TSC1* or *TSC2* (9%), and overexpression of *AKT3* (10%). Alterations in the receptor tyrosine kinase/RAS pathway included activation of *FGFR3* (17%), amplification of *EGFR* (9%), mutations of *ERBB3* (6%), and mutation or amplification of *ERBB2* (9%).

The TCGA expanded cohort analysis on 412 MIBC that identified 58 significantly mutated genes and confirmed the high mutation rate of MIBC [46]. Moreover, RNA expression analysis identified five expression subtypes that may stratify response to different treatments: luminal-papillary (35%), luminal (6%), basal-squamous (35%), luminal-infiltrated (19%), and neuronal (5%) [46]. Recently, a consensus molecular classification of MIBC has been proposed on the basis of 1750 MIBC transcriptomic profiles from 18 datasets comparing six molecular classification schemes. Six molecular classes were identified: luminal papillary, luminal nonspecified, luminal unstable, stroma-rich, basal/squamous, and neuroendocrine-like [47]. This consensus classification has possible therapeutic implications. In fact, the different consensus classes are associated with different stromal components and genetic alteration that could possibly identify a subset of patients more likely to respond to immunotherapy or to target therapy. The identification of molecular alterations is of great importance since many target therapies are being studied for the management of advanced UT [48].

FGFR1, FGFR2, FGFR3, FGFR4 are tyrosine kinases receptor that have been found altered in UC [49]. Activating FGFR3 mutations are most common in NMIBC, being identified in approximately two-third of these early stage tumors, while their frequency in MIBC is lower (less than 25%), including amplifications, mutations, and fusions in FGFR gene [50,51,52,53]. The activating FGFR3 mutation leads to ligand-independent receptor dimerization and constitutive downstream signal transduction [53]. The presence of activating point mutations in FGFR3 in early stage tumors is associated with favorable outcome [54]. Approximately 7% of UC present an amplification of FGFR1 [55]. FGFR1 has two splicing variants, FGFR1α and FGFR1β, that are equally expressed in normal urothelium, but the FGFR1β variant is predominant in UC and its expression correlates with tumor grade and stage [56]. The luminal-papillary subtype of the consensus classification is characterized by a high rate of FGFR3 mutations and translocations, suggesting that these tumors may respond to FGFR inhibitors [47]. Moreover, FGFR3 pathway was found to be activated in non-T-cell-inflamed tumors that are likely to present intrinsic resistance to ICIs [57]. Furthermore, immunotherapy seems to be less effective on TCGA luminal I subtype also based on an exploratory analysis of a phase 2 trial: luminal I cluster presented lower expression levels of CD8+ genes, lower PD-L1 immune cell or tumor cell expression, and lower responses to the anti-PD-L1 atezolizumab [38].

With these premises, multi-tyrosine kinase inhibitors targeting FGFR alterations have been studied in patients with metastatic UC [58]. The results of a phase 2 trial (BLC2001) testing the tyrosine kinase inhibitor of FGFR1–4 erdafitinib have been recently published [59]. In this trial, 99 patients with locally advanced or metastatic UC with FGFR3 mutation or FGFR2/3 fusion and progressed to at least one previous chemotherapy or treatment naïve if cisplatin ineligible were assigned to receive erdafitinib, 8 mg per day in a continuous regimen. The primary endpoint of the study was ORR. The treatment was found to be active with an ORR of 40% (3% with a complete response and 37% with a partial response). The median duration of progression-free survival (PFS) was 5.5 months and the median duration of OS was 13.8 months. Interestingly, the 22 patients previously treated with ICIs presented a response rate of 59%. Grade 3 or higher treatment-related adverse events were reported in nearly half the patients and the most common of any grade were hyperphosphatemia, stomatitis, diarrhea. FDA granted accelerated approval to erdafitinib for patients with FGFR3 or FGFR2 genetic alterations progressed during or following platinum-containing chemotherapy, including within 12 months of neoadjuvant or adjuvant platinum-containing chemotherapy.

Another pathway implicated in UC pathogenesis and progression is vascular endothelial growth factor receptors (VEGFR) 1 and 2 and their ligands (vascular endothelial growth factor, VEGF-A, -B, -C, and -D) [60,61]. Angiogenesis by microvessel quantification resulted to be an independent predictor of survival in patients with invasive bladder cancer and serum levels of VEGF have been correlated with tumor stage and grade, vascular invasion and presence of metastases [62,63,64].

VEGF/VEGFR inhibitors as single agents or in combination with chemotherapy have been investigated for the treatment of advanced UC. Single agent treatment with sorafenib, pazopanib, cabozantinib and sunitinib resulted to have limited activity and limited effect on clinical outcomes [65,66,67,68]. Similarly, combination therapies failed to be shown to be more active than chemotherapy alone: vandetanib combined with docetaxel or sunitinib associated with gemcitabine and cisplatin did not improve clinical activity and were more toxic [69,70]. 

The monoclonal antibody against VEGF Bevacizumab was evaluated in a phase II trial in association with gemcitabine and cisplatin in first line of therapy for metastatic UC: the combination treatment showed an ORR of 72% and an OS of 19.1 months [71]. Unfortunately, the subsequent phase III trial (CALGB-90601 Alliance) failed to show an advantage in OS, the primary endpoint of the study, for the combination regimen [72].

A phase III randomized trial investigated the combination of ramucirumab plus docetaxel versus placebo plus docetaxel in 530 patients with advanced or metastatic UC progressed during or after platinum-based chemotherapy [73]. The experimental arm was associated with a significantly longer PFS (4.07 months versus 2.76 months in the docetaxel-alone arm) with no OS benefit. Additional follow-up confirmed the advantage in PFS (4.1 months versus 2.8 months in the experimental arm versus the control arm, respectively; HR 0.696; *p* = 0.0002) and the lack of statistically significant advantage in OS for the combination treatment (9.4 months in the experimental arm versus 7.9 months in the placebo group; stratified HR 0.887; *p* = 0.25) [74].

### 2.3. Antibody-Drug Conjugates 

Another interesting emerging class for the treatment for metastatic UC is antibody-drug conjugate (ADC), that consists in monoclonal antibody against a target expressed on cancer cell bounded to a cytotoxic agent with a protease-cleavable or non-cleavable linker [75]. When the monoclonal antibody binds to a tumor antigen, the drug is internalized and the active chemotherapeutic agent is released into the selected cells, leading to cell death. This mechanism of cell-killing is supposed to limit exposure and toxicity of cytotoxic agents. One of the most promising antibody-drug conjugate currently under investigation in metastatic UC is enfortumab vedotin (ASG-22ME). This ADC is composed of an anti nectin-4 (a cell adhesion molecule highly expressed in UC) monoclonal antibody liked to a micro-tubule-disrupting agent (monomethyl auristatin E). The phase 1 EV-101 trial evaluated enfortumab vedotin in patients with Nectin-4-expressing solid tumors, including 155 heavily pretreated patients with metastatic UC [76]. Single-agent enfortumab vedotin resulted to be well tolerated and showed clinically meaningful and durable responses with an ORR of 43%, a duration of response of 7.4 months, a median OS of 12.3 months, and OS rate at 1 year of 51.8%. 

The phase II EV-201 single-arm study investigated enfortumab vedotin in locally advanced or metastatic UC patients previously treated with ICI and platinum-containing chemotherapy (Cohort 1) or an ICI and no prior chemotherapy (Cohort 2) [77]. The preliminary data of cohort 1 enrolling 128 patients have been presented and showed an ORR of 42% with 9% complete responses. The safety profile was manageable with fatigue (50%), alopecia (48%), and decreased appetite (41%) as most common treatment-related adverse events. Of note, one death was reported as treatment related by the investigator (interstitial lung disease). Based on these results, the FDA granted accelerated approval to enfortumab vedotin for patients with locally advanced or metastatic urothelial cancer who have previously received a PD-1/PD-L1 inhibitor and a platinum-containing chemotherapy in the neoadjuvant/adjuvant, locally advanced or metastatic setting. A phase III trial evaluating enfortumab vedotin in patients progressed to previous ICI and platinum containing chemotherapy is ongoing (NCT03474107, EV-301).

Preliminary data for the combination of enfortumab vedotin with pembrolizumab for first line treatment of cisplatinum ineligible patients with metastatic UC are encouraging. The phase Ib study EV-103 (NCT03288545) demonstrated the efficacy of this combination approach in this subset of patients with a tolerable and manageable safety profile [78,79]. At the recent 2020 American Society of Clinical Oncology (ASCO) Genitourinary Cancer Symposium, the updated results were presented by Rosenberg: at a median follow-up of 11.5 months, investigator-assessed objective response rate was confirmed to be 73.3%, with 15.6% complete responses [80]. The most common adverse events were fatigue (58%), alopecia (53%), and neuropathy (53%). A phase III trial with this combination therapy is ongoing (NCT04223856, EV-302).

Another ADC that has been evaluated in metastatic UC is Sacituzumab govitecan, a humanized anti-Trop-2 (an epithelial cell surface antigen overexpressed in UC) monoclonal antibody linked with SN-38 (the active metabolite of irinotecan). Sacituzumab govitecan has been investigated in a phase I/II basket study in 45 patients progressed after at least one prior systemic therapy [81]. The ORR was 31%, including 2 CR and 12 PR. In patients with visceral involvement the ORR was 27% and in patients previously treated with ICIs it was 23%. Median PFS and OS were 7.3 months and 18.9 months, respectively. Among grade ≥3 adverse events there were neutropenia/neutrophil count decreased (38%), anemia (11%), hypophosphatemia (11%), diarrhea (9%), fatigue (9%), and febrile neutropenia (7%). A global, single-arm, phase II trial which is ongoing (TROPHY-U-01, NCT03547973) is evaluating the antitumor activity of Sacituzumab govitecan (10 mg/kg, days 1 and 8 of 21-day cycles) in patients with advanced UC. Cohort 1 [82] assessed the activity in 35 patients progressed to platinum-based regimens and ICIs while cohort 2 [83] enrolled 18 platinum-ineligible patients who progressed after first-line ICI. The interim results of cohort 1 demonstrated an ORR of 29% with 2 confirmed CR, 5 confirmed PR, and 3 unconfirmed PR. The preliminary results of cohort 2 showed an ORR of 28% with 4 confirmed PRs, and 1 PR pending confirmation. The safety profile was consistent with prior reports in both cohorts no treatment-related deaths were reported.

**Figure 3:**
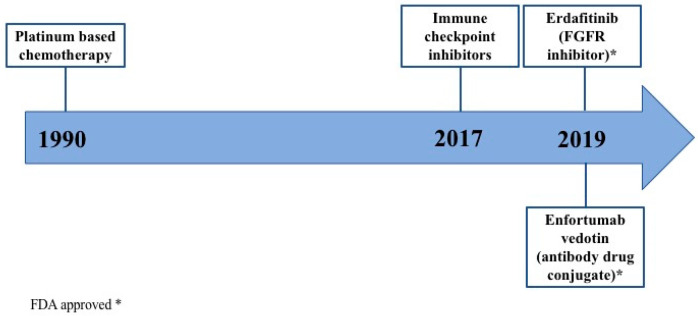
Evolution of practice changing treatments for metastatic urothelial carcinoma. For many years, platinum-based chemotherapy has been the gold standard treatment for patients with metastatic urothelial carcinoma. Since 2017, immune checkpoint inhibitors entered in the treatment scenario. In 2019, two new treatment strategies showed promising results and have been granted accelerated approval by the US Food and Drug Administration: erdafitinib and enfortumab vedotin.

**Figure 4:**
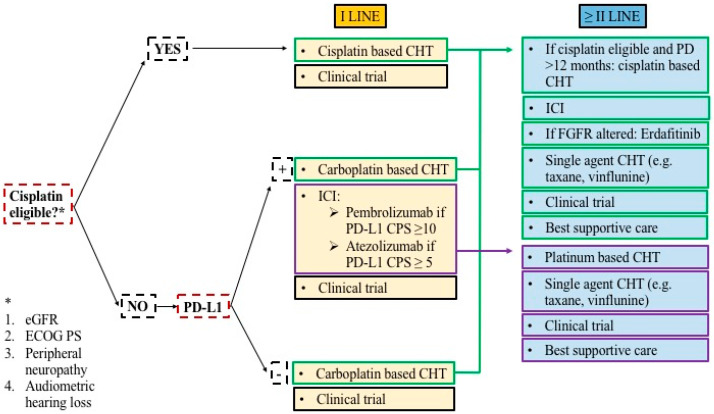
Current treatment algorithm for metastatic urothelial carcinoma. Based on cisplatin eligibility and PD-L1 positivity, patients are currently being treated as indicated in the figure. If cisplatin eligible (depending on eGFR, ECOG PS, peripheral neuropathy, audiometric hearing loss) patients should be treated with cisplatin-based chemotherapy. In cisplatin ineligible patients, the treatment changes according to PD-L1 positivity. If PD-L1 negative, patients should be treated with carboplatin-based chemotherapy. If PD-L1 positive, carboplatin-based chemotherapy or immunotherapy are the available options. Second and later lines of treatment depend on previous exposure to chemotherapy or immunotherapy. Enrollment in clinical trials should always be considered as a treatment option. CHT: chemotherapy. ICI: immune checkpoint inhibitor. PD: progressive disease. FGFR: fibroblast growth factor receptor. CPS: combined positive score. PD-L1: programmed death ligand-1. eGFR: estimated glomerular filtration rate. ECOG PS: eastern cooperative oncology group performance status.

The evolution of practice changing treatments, including promising therapies approved by FDA, for metastatic UC is depicted in Figure 3. Current treatment scenario in metastatic UC is reported in Figure 4.

## 3. Therapeutic Approaches UNDER Evaluation

### 3.1. Ongoing Trials Evaluating ICIS

#### 3.1.1. Combination of ICIs with Cytotoxic Chemotherapy

Several trials exploring the role of ICIs plus chemotherapy in different settings and combinations are currently ongoing. As regards the first-line setting, the randomized phase III KEYNOTE-361 trial (NCT02853305) is investigating the safety and efficacy of front-line pembrolizumab with or without chemotherapy (GC in eligible patients or gemcitabine–carboplatin combination in cisplatin unfit subjects) [84]. OS and PFS are the primary endpoints of this study, with ORR and safety assessed as secondary endpoints. Similarly, the phase III IMvigor130 trial (NCT02807636) has enrolled previously untreated patients affected by advanced or metastatic UC in a 1:1:1 ratio to either atezolizumab plus platinum-gemcitabine, atezolizumab monotherapy, or platinum-gemcitabine plus placebo [85]. The primary endpoints are OS and PFS; secondary endpoints are safety, ORR and DCR. As previously stated, preliminary findings of these two trials showing a close relation between PD-L1 expression, type of treatment and clinical outcomes have had a relevant impact on current indications of pembrolizumab and atezolizumab in UC. More specifically, FDA revised previous indications for the two ICIs, which are now limited for (1) first-line treatment in cisplatin-ineligible patients whose tumors express PD-L1 (CPS ≥ 10% for pembrolizumab and PD-L1 stained tumor-infiltrating immune cells covering ≥ 5% of the tumor area in the case of atezolizumab), (2) subjects which have disease progression during or following platinum-containing chemotherapy, or (3) patients unfit for any platinum-based chemotherapy, regardless of PD-L1 expression.

The anti-PD-L1 agent atezolizumab is being also investigated in a phase II trial (NCT03093922) aimed to evaluate the safety and efficacy of two different dosing schedules of atezolizumab in combination with GC as front-line treatment for advanced or metastatic UC. Regarding less commonly used ICIs, a randomized, placebo-controlled, phase III trial (NCT03967977) has been initiated to investigate the safety and efficacy of front-line tislelizumab plus standard chemotherapy (gemcitabine plus either cisplatin or carboplatin) versus placebo plus standard chemotherapy (gemcitabine plus either cisplatin or carboplatin). Tislelizumab (BGB-A317) is a humanized monoclonal PD-1 antibody which is being evaluating in several solid tumors [86]. 

With regard to second-line setting, atezolizumab is currently under evaluation in cisplatin-ineligible patients in an ongoing phase II trial (NCT03737123). In this study, subjects who previously received sequential or concurrent ICI and carboplatin-based chemotherapy will be treated with atezolizumab plus docetaxel combination; conversely, patients who have already received an ICI without prior platinum-based chemotherapy will be treated with atezolizumab plus carboplatin-gemcitabine. 

Another anti-PD-L1 agent, avelumab, is being investigated in phase II trial on previously untreated, cisplatin-ineligible patients (NCT03390595). In this study, patients are randomized in a 1:1 ratio to receive avelumab in combination with carboplatin-gemcitabine chemotherapy versus carboplatin-gemcitabine alone. Avelumab is also under investigation in a phase II trial comparing avelumab plus GC versus GC in cisplatin fit, treatment naïve patients (NCT03324282). PT-112, a platinum-based agent belonging to the phosphaplatin family, is under evaluation in combination with avelumab in the ongoing phase I/II PAVE-1 trial (NCT03409458). 

The combination of the anti-PD-1 antibody pembrolizumab with paclitaxel is currently under investigation in a phase II trial (NCT02581982) on platinum-refractory patients. Lastly, several other combinations of anti-PD-1/PD-L1 agents with cytotoxic agents such as pemetrexed, platinum, and etoposide are being evaluated in a series of ongoing trials (NCT03744793; NCT03582475).

Ongoing phase I/I/II trials, either recruiting or active not recruiting, of ICIs in combinations with cytotoxic chemotherapy in advanced or metastatic UC are summarized in Table 1.

#### 3.1.2. Combination of ICIs with Other ICIs

In recent years, checkpoint-inhibition combination therapies have provided outstanding efficacy gains in several malignancies including melanoma, lung cancer and renal cell carcinoma [87,88]. The underlying rationale for these combinations lies in the synergistic effect provided by the inhibition of CTLA-4 and PD-1/PD-L1, resulting in an enhance of T-cell function through distinct pathways [89]. The results obtained in a number of cancer types have led to the recent attempt to translate these experiences in advanced or metastatic UC.

The phase 1/2 CheckMate-032 trial investigated ipilimumab plus nivolumab versus nivolumab alone in several malignancies, including platinum-refractory patients affected by advanced or metastatic UC [90,91]. In this cohort of subjects, the combination of the two immunotherapies yielded a promising response rate of 38%; moreover, subjects treated with the combination showed a median OS of 15.3 months versus 9.9 months in the nivolumab arm. The combination of an anti-PD-1 and a CTLA-4 antibody is being investigated also in the CheckMate-901 trial (NCT03036098) [92], aimed to evaluate the efficacy of nivolumab ± ipilimumab versus GC or carboplatin-gemcitabine chemotherapy. The same combination with modified schedules and additional nivolumab/ipilimumab “boost” cycles is under evaluation also in a Phase II trial (NCT03219775, TITAN-TCC) on treatment naïve and platinum-refractory patients with advanced or metastatic UC.

The anti-PD-L1 agent durvalumab, registered by FDA as monotherapy in previously treated patients affected by advanced or metastatic UC, is currently under investigation in combination with the CTLA-4 IgG2-kappa monoclonal antibody tremelimumab in the DANUBE (NCT02516241) and the NILE (NCT03682068) trials [93,94]. The DANUBE is an ongoing randomized, open-label, phase III trial aimed at ascertaining the value of front-line durvalumab ± tremelimumab versus platinum-gemcitabine chemotherapy in advanced or metastatic UC [89]. In the same setting of previously untreated patients, the NILE trial randomized subjects to three different cohorts: durvalumab plus tremelimumab plus platinum-gemcitabine; durvalumab plus platinum-gemcitabine; platinum-gemcitabine [94]. 

Ongoing phase II/III trials, either recruiting or active not recruiting, of ICIs in combinations with other ICIs in advanced or metastatic UC are summarized in Table 2.

#### 3.1.3. Combination of ICIs with Antiangiogenic Agents

Given the importance of angiogenesis as a crucial process in the carcinogenesis and progression of UC, ICIs are under evaluation also in combination with VEGFR antibodies and TKIs, including bevacizumab, ramucirumab, lenvatinib, and several others [61,95]. 

As regards front-line treatment, the VEGF-A monoclonal antibody bevacizumab is being investigated in a phase II trial assessing bevacizumab plus atezolizumab in treatment-naïve, cisplatin-ineligible patients (NCT03272217). 

Axitinib, a highly selective VEGFR-1, -2, and -3 inhibitor, is currently under investigation as front-line treatment in combination with avelumab in the ongoing phase II trial JAVELIN Medley VEGF (NCT03472560). Enrolled subjects are deemed ineligible for receiving cisplatin-containing first-line chemotherapy and the primary endpoint is ORR, defined as a confirmed CR or PR. 

Ramucirumab is an IgG1 monoclonal antibody that binds VEGFR-2 preventing ligand binding and receptor-mediated pathway activation in endothelial cells [96]. An ongoing, phase I trial is assessing the safety of ramucirumab in combination with pembrolizumab in previously treated patients affected by a number of solid cancers, including UC (NCT02443324). 

Lenvatinib is a small TKI able to inhibit VEGFR-1, FGFR1–4, stem cell factor receptor (KIT), platelet-derived growth factor receptor α (PDGFRα), and rearranged during transfection (RET) [97]. The combination of lenvatinib and pembrolizumab is being investigated as front-line treatment in the phase III LEAP-011 trial (NCT03898180) which is evaluating the combination in cisplatin-unfit subjects with PD-L1 CPS ≥10 or in patients deemed ineligible for any platinum-based regimen, regardless of PD-L1 expression. 

Cabozantinib is another small TKI inhibiting a plethora of targets which play an important role in tumor growth, angiogenesis, and survival, such as VEGFR-2, MET, RET, KIT, AXL, and FLT3 [98,99]. Following the findings of a recent phase I trial where cabozantinib plus nivolumab plus ipilimumab yielded an ORR of 36% across all genitourinary cancers [100], this molecule is being evaluated in combination with pembrolizumab (NCT03534804), durvalumab (NCT03824691), atezolizumab (NCT03170960), and nivolumab plus ipilimumab (NCT03866382) in treatment-naïve and previously treated patients. Moreover, cabozantinib is also under investigation in a phase II trial (NCT04066595) which is enrolling previously treated subjects with platinum-based chemotherapy (cohort 1) and platinum-based chemotherapy plus ICIs (cohort 2).

The anti-VEGF recombinant EphB4-HSA fusion protein is currently under evaluation in combination with pembrolizumab in an ongoing phase II trial (NCT02717156). The study is enrolling treatment naïve patients affected by locally advanced or metastatic UC. 

Apatinib, a small-molecule TKI which selectively inhibits VEGFR-2 resulting in a decrease in endothelial proliferation, migration, and tumor microvascular density, is under evaluation in combination with pembrolizumab in a phase I/IIa trial (NCT03407976; APPEASE). In this study, eligible subjects must have progressed during or following platinum-based chemotherapy. 

Lastly, sitravatinib, a small TKI able to inhibit VEGFR, PDGFR, KIT, RET, and MET [101], is currently under investigation in combination with nivolumab in a non-randomized, Phase II trial (NCT03606174). Although all patients are planned to receive the same treatment (nivolumab 240 mg every 2 weeks or 480 mg every 4 weeks plus sitravatinib 120 mg orally once per day continuously in 28-day cycles), eligible subjects are assigned to eight different cohorts, based upon previous therapies for UC.

Ongoing phase I/II/III trials, either recruiting or active not recruiting, of ICIs in combinations with antiangiogenic agents in advanced or metastatic UC are summarized in Table 3.

#### 3.1.4. ICI Monotherapy

Although combination therapies are displaying the ability to broaden the anticancer activity of ICIs and the majority of ongoing trials are testing ICIs in combination with other anticancer agents, some trials are evaluating the role of monotherapy in different settings. 

The anti-PD-1 agent pembrolizumab is being evaluated in a randomized, double-blinded phase II trial (NCT02500121) assessing the role of maintenance pembrolizumab (200 mg flat dose every three weeks, for up to 24 months) versus placebo after front-line chemotherapy in patients affected by metastatic UC. Eligible subjects must have achieved CR, PR or stable disease (SD) after 4 to 6 cycles of first-line platinum-based chemotherapy; six-month PFS assessment, regardless of PD-L1 expression, is the primary outcome. Maintenance treatment with ICIs is also under investigation in an ongoing phase III trial (NCT02603432) comparing avelumab maintenance plus best supportive care versus best supportive care alone in patients whose disease did not progress after first-line platinum-based chemotherapy. 

Atezolizumab treatment is being tested in the real-world phase III SAUL trial (NCT02928406) and preliminary results of this study assessing the role of atezolizumab in a pretreated population of 1004 UCs have been recently published [102]. Median OS and PFS were 8.7 and 2.2 months respectively, with an ORR of 13%. The trial enrolled patients who experienced progression during or after one to three prior therapies, of which 10% had ECOG-PS 2 and 98% were platinum pretreated. 

Toripalimab (JS001), a recombinant, humanized PD-1 monoclonal antibody capable of preventing the binding of PD-1 with PD-L1 and PD-L2, is being evaluated as monotherapy in pretreated advanced or metastatic UC in an ongoing phase II trial (NCT03113266). The primary outcome is ORR, with duration of response, PFS, OS, and safety as secondary outcomes. 

The anti-CTLA-4 agent tremelimumab is currently being evaluated as monotherapy in a phase II trial (NCT03557918) assessing ORR in patients with metastatic UC which previously received PD-1/PD-L1 blockade. 

The novel anti-PD-L1 CK-301 (Cosibelimab) is being tested in a phase I trial (NCT03212404) on a number of advanced malignancies, including UC. Lastly, a phase I trial (NCT03053466) is studying the role of the anti-PD-1 agent APL-501 in patients affected by advanced solid tumors presenting at least 1% of PD-L1 expression by IHC.

Ongoing phase I/II/III trials, either recruiting or active not recruiting, of ICI monotherapy in advanced or metastatic UC are summarized in Table 4.

#### 3.1.5. Novel Immunotherapy Approaches

With the aim to enhance the response to ICIs and other anticancer agents, a number of novel immunomodulatory molecules and brand-new combinations are being evaluated in UC [103,104]. 

A recently emerging immunotherapeutic target is represented by the indoleamine 2,3-dioxygenase-1 (IDO1), an enzyme playing a crucial role in immunosuppression, angiogenesis, and metastasis [105]; in fact, IDO1 is an immune regulatory enzyme which promotes tryptophan depletion, a mechanism necessary for T-cell survival [106]. More specifically, IDO1 enhances the activity of CD4+ T regulatory cells and myeloid-derived suppressor cells and, conversely, is able to suppress CD8+ T effector and natural killer (NK) cells [107]. Despite early promising results, the combination of pembrolizumab plus the IDO-1 inhibitor epacadostat came up short against its primary endpoints of OS and PFS; thus, the two trials assessing the role of the anti-IDO-1 ± pembrolizumab in treatment-naïve, cisplatin ineligible subjects (NCT03361865) and in platinum-refractory patients (NCT03374488) arrested recruitment. Currently, the safety of the combination of pembrolizumab plus KHK2455, a long-active selective IDO-1 inhibitor, is being evaluated in an ongoing Phase I study on platinum-refractory patients affected by metastatic UC (NCT03915405).

Another attracting target is represented by the tumor necrosis factor receptor OX40 (CD134) [108,109]; when activated by its ligand OX40L, OX40 is involved in T-cell signaling activation, promoting T-cell survival and enhancing the expression of several molecules such as Bcl-2 anti-apoptotic molecules, cytokines, cyclin A, and cytokine receptor [110]. Therefore, as OX40 may promote proliferation and survival of CD4+ and CD8+ T cells, immunostimulatory agonistic agents are currently under investigation in several solid malignancies [111]. The OX40 agonist PF-04518600 is being evaluated as monotherapy or in combination with the cytokine modulator utomilumab (PF-05082566)—a monoclonal antibody with agonist activity toward 4-1BB (CD137), a receptor expressed on NK, CD8+, and CD4+ T cells [112]. Preliminary results of this trial, which includes also a cohort of patients affected by UC, have shown an ORR of 5.4% across all cancer types; nevertheless, the promising 50% of ORR reported in the UC subgroup has led to the NCT03217747 and the Javelin Medley (NCT02554812) ongoing phase I/II trials which are evaluating the OX40 agonist PF-04518600 in combination with ICIs, radiation therapy, utomilumab, and cytotoxic chemotherapy. Finally, the hexavalent OX40 agonist INBRX-106 is currently under investigation as monotherapy or in combination with pembrolizumab for previously treated patients in a phase I trial (NCT04198766).

Other immunotherapeutic strategies currently under investigation include cytokine agonists such as NKTR-214 (bempegaldesleukin)—an IL-2 pathway agonist which targets CD122, a protein expressed in NK and CD8 T cells—ALT-803 and YT107 [113,114]. Following promising early results from a Phase I trial across several solid tumors (NCT02983045, PIVOT-02), NKTR-214 is currently being evaluated in combination with nivolumab in treatment-naïve, cisplatin ineligible patients affected by locally advanced or metastatic UC (NCT03785925, PIVOT-10). Conversely, NKTR-214 is now being investigated in the phase I PROPEL trial (NCT03138889) assessing the combination of atezolizumab plus NKTR-214 in platinum-refractory UC. Similarly, the recombinant human interleukin-7 CYT-107 is under evaluation in combination with atezolizumab versus atezolizumab alone in platinum-refractory UCs (NCT03513952); the IL-15 superagonist ALT-803 is being investigated as combination therapy with pembrolizumab, nivolumab, atezolizumab, or avelumab in previously treated patients (NCT03228667). Finally, an ongoing open-label, non-randomized phase I study (NCT03809624) is testing the role of INBRX-105 in advanced solid tumors. INBRX-105, a next generation bispecific antibody targeting PD-L1 and 4-1BB, blocks inhibitory PD-1/PD-L1 axis and simultaneously activates essential co-stimulatory activity via 4-1BB. Other bispecific antibodies such as GEN1046, XmAb20717, XmAb22841, and XmAb23104 are currently under investigation in ongoing phase I (NCT03752398, NCT03849469, NCT03517488) and phase I/II (NCT03917381) trials.

Another potential target is lymphocyte activation gene-3 (LAG-3, CD223), a co-inhibitory receptor able to suppress T-cell activation and cytokines secretion [115]; more specifically, LAG-3 overexpression in tumor cells is involved in the phenomenon of immune exhaustion, with suppression of T-cell function [116,117]. Thus, LAG-3 inhibitors as monotherapy or in combination with anti-PD-1 agents are currently being explored in several phase I and II trials in advanced malignancies, including pretreated UC (NCT03538028, NCT03250832).

T-cell immunoglobulin and mucin-domain containing-3 (TIM-3) is another co-inhibitory receptor expressed on regulatory T cells, effective T cells, tumor cells, and innate immune cells (macrophage and dendritic cells) [118]. TIM-3 expression has been recently associated with poor prognosis in a number of cancer types, including UC [119,120,121]; because of the implication of TIM-3 overexpression in T-cell dysfunction and exhaustion, several TIM-3 inhibitors are currently being studied in advanced cancer. Among them, INCAGN02390 is under evaluation in a phase I trial (NCT03652077) assessing its role as monotherapy in previously treated metastatic malignancies including UC. 

Glucocorticoid-induced TNF receptor family related receptor (GITR) represents a co-stimulatory receptor that binds the GITR ligand (GITRL) [122]; the activation of GITR can result in signals influencing the activity of CD4+, CD8+ and regulatory T cells, playing an important role in autoimmune and inflammatory diseases as well as in anticancer immune response [123,124]. Thus, GITR seems to be a promising target for novel immunotherapy agents. A phase I/II trial analyzing the combination of nivolumab, ipilimumab, and the GITR agonist INCAGN01876 (NCT03126110) in patients with metastatic malignancies including UC is recruiting at present. 

Chimeric antigen receptor (CAR)-T immunotherapy has shown impressive responses in a number of B cell malignancies and is currently being tested in several solid tumors, including advanced or metastatic UC (NCT03185468) [125]. CAR-T action is based on engineered T cells expressing a CAR; current second-generation CAR are receptors composed of (1) an extracellular, epitope-specific binding domain, (2) a transmembrane domain, (3) and an intracellular domain of the T cell receptor; this last domain consists in its turn of costimulatory molecules such as CD28, 4-1BB, and the CD3ζ chain, and is involved in a massive activation of T cells which is independent from T-cell receptor (TCR)—major histocompatibility complex (MHC) interactions [126,127].

Lastly, another promising immunotherapeutic strategy lies in tumor vaccines (TVs), which are currently under investigation in many solid tumors [128,129]. As regards UC, the majority of developing TVs concerns BCG-relapsing, non-muscle invasive disease, where neo-antigens are being studied in combination with immune-stimulating adjuvant agents, cytotoxic agents, and/or mTOR inhibitors (NCT01353222, NCT02015104, NCT01498172). Cancer vaccines are also under investigation in combination with ICIs, as in the case of the NCT03689192 and the NCT03639714 trial. In the NCT03639714 Phase 1/2 Study, two vaccines vectors (GRT-C901 and GRT-R902)—used as immune boosts—are being investigated in combination with nivolumab plus ipilimumab in patients affected by a number of solid cancers including previously treated, metastatic UC.

Ongoing phase I/II/III trials, either recruiting or active not recruiting, of novel immunotherapy approaches in advanced or metastatic UC are summarized in Table 5.

### 3.2. PARP Inhibitors

One of the new promising therapeutic approaches is the use of Poly(ADP-ribose) polymerase (PARP) inhibitors that target DNA repair gene mutations and have been proven active in other type of cancer like ovarian, breast, and prostate cancer [130,131].

Regarding UC, genomic alterations in DNA repair genes like *ATM, ERCC2, RAD51B* were found in 2–14% and in *BRCA 1/2, PALB2, FANCD2, ERCC2, ATM* in 3.7–12.3% of MIBC [46,132]. Moreover, patients with DNA damage response and repair (DDR) gene alterations treated with platinum based chemotherapy resulted to have better PFS and OS [133]. In fact, in multiple tumors the presence of DDR gene aberrations correlates with an enhanced sensibility to platinum compounds [134]. Based on these results, PARP inhibitors have been studied in UC as well [135,136,137,138]. At the recent ASCO Genitourinary Cancers Symposium 2020, the results of the study ATLAS (NCT03397394) were presented [134]. This phase II trial assessed the efficacy and safety of the PARP inhibitor rucaparib in 97 patients with locally advanced or metastatic UC with or without homologous recombination deficiency (HRD), progressed to one or two prior treatments. Total of 20.6% of patients were HRD-positive, 30.9% were HRD-negative, and 48.5% had unknown HRD status. Among patients with sequencing results (64 patients), deleterious alterations in *BRCA1*, *BRCA2*, *RAD51C*, *PALB2* were infrequent (9.4%). Common alterations were found in *TP53* (52.4%) and in *FGF*/*FGFR* pathway (77.6%). The results showed that there were no confirmed responses to rucaparib, 28.1% of patients achieved a stable disease as best response with no difference in efficacy between HRD-negative and HRD-positive patients. The trial was discontinued because protocol-defined continuance criteria were not meet. Two phase II trials are investigating the PARP inhibitor olaparib in monotherapy in chemotherapy naïve cisplatin ineligible patients or progressed to first line treatment selected for DDR mutations (NCT03448718) and in patients with DNA-repair defects progressed to 1 or 2 prior treatment regimens (NCT03375307).

A phase II trial is currently investigating the PARP inhibitor niraparib as maintenance therapy until disease progression or unacceptable toxicity or death in patients unselected for DDR mutational status not progressing to first line platinum-based chemotherapy (NCT03945084).

Another strategy being tested is combination therapy of PARP inhibitors with ICIs or target therapies.

Indeed, the presence of alteration in DDR genes has been associated with higher mutational load and higher response to ICIs in patients with UC [139,140]. Based on these observations, several combinations of PARP inhibitors and PD-1/PD-L1 inhibitors are currently being tested: durvalumab plus olaparib (module B, NCT02546661, active not recruiting; NCT03459846, active not recruiting), rucaparib plus nivolumab (NCT03824704, active not recruiting), niraparib plus atezolizumab (NCT03869190, recruiting).

The phase I BISCAY (NCT02546661) trial is evaluating the combination of durvalumab with olaparib or a FGFR1-3 inhibitor (AZD4547) or a TORC 1 and 2 inhibitor (vistusertib) in platinum refractory, immuno-therapy naïve UC patients allocated depending on tumour DNA alterations determined by next generation sequencing. Total of 391 patients were screened and NGS analysis showed the following absolute frequency of biomarkers: *FGFR1–3* fusions or *FGFR3* activating mutations in 21% of cases (83 patients in the AZD4547 arm/391), HRR deleterious gene alterations (*ATM, BARD1, BRCA1, BRCA2, BRIP1, CDK12, CHEK1, CHEK2, FANCI, FANCL, PALB2, RAD51B, RAD51C, RAD51D, RAD54L*) in 14% of cases (54 patients in the olaparib arm/391), *RICTOR* amplification and *TSC1/TSC2* loss or inactivating mutations in 15% cases (60 patients in the Vistusertib arm/391). The preliminary results available on 14 patients with homologous recombination repair genomic alterations treated with olaparib and durvalumab showed a high tumor mutation burden and a confirmed ORR of 35.7%, a 6-months PFS rate of 42%, 1-years OS rate of 54% [141].

A phase Ib-II trial (NCT03992131) is evaluating the combination between the PARP inhibitor rucaparib and lucitanib, a VEGFR1-2-3, FGFR1-2, and PDGFRα-β inhibitor, or sacituzumab govitecan.

Ongoing phase I/II/III trials, either recruiting or active not recruiting, of PARP inhibitors in advanced or metastatic UC are summarized in Table 6.

### 3.3. Target Therapy

As already discussed, the FGFR inhibitor erdafitinib is a promising treatment strategy in patients with FGF/FGFR alterations. In these subgroup of patients, other therapies directed at inhibiting FGFR are currently being tested: PRN1371, a FGFR 1-4 inhibitor, in a phase I trial in previously treated patients (NCT02608125); Pemigatinib, a FGFR1-3 inhibitor, in phase II trial in patients progressed to at least one prior treatment (NCT02872714, FIGHT-201); Rogaratinib (BAY1163877), a FGFR 1-4 inhibitor, in a phase II/III trial in patients progressed to at least one platinum-containing regimen (NCT03410693).

Moreover, FGFR inhibitors are being evaluated in combinations with PD-1/PD-L1 inhibitors. A study by Sweis et al. showed that FGFR3 pathways were activated in non-T-cell-inflamed UC, characterized by an absence of intratumoral T cells, thus identifying a potential targetable pathway that could help to overcome tumor-intrinsic immunotherapy resistance [57]. The updated results of the interim analysis of the phase II study FIERCE-22 (NCT03123055) evaluating the combination of the FGFR3 inhibitor vofatamab (a human IgG1 monoclonal antibody directed against FGFR3) in combination with pembrolizumab in 28 patients (20 wild-type) progressed following platinum-based chemotherapy have been presented at European Society for Medical Oncology (ESMO) Congress 2019: the combination therapy resulted to be well tolerated with encouraging ORR (29.6%) and a median PFS is 4.7 months [142].

Combination of FGFR inhibitors and ICIs currently under investigation in ongoing clinical trials are: Rogaratinib ± atezolizumab in first-line treatment of cisplatin ineligible patients with FGFR 1-3 alterations (NCT03473756, FORT-2); pemigatinib ± pembrolizumab versus standard of care (chemotherapy or pembrolizumab) in first-line treatment cisplatin-ineligible patients with FGFR3 mutation or rearrangement (NCT04003610, FIGHT-205); derazantinib (a FGFR 1-3 inhibitor) ± atezolizumab in cisplatin ineligible patients with FGFR alteration in first-line or progressed to prior FGFR inhibitor treatment (NCT04045613); erdafitinib plus cetrelimab (an IgG4 monoclonal antibody directed against PD-1) in pretreated (phase Ib) or previously untreated cisplatin-ineligible patients (phase II) (NCT03473743).

Other target therapy treatments under evaluation are PI3K/mTOR inhibitors since this pathway resulted to be frequently altered in UC, as already discussed [45]. mTOR inhibitors and PI3K inhibitors are currently being tested alone (everolimus: NCT00805129, sapanisertib: NCT03047213, Buparlisib: NCT01551030), and in combination with anti-PD-1/PD-L1 (nivolumab plus nab-rapamycin: NCT03190174, durvalumab plus vistusertib: NCT02546661, module E) or chemotherapy (paclitaxel plus TAK-228: NCT03745911).

Another interesting pathway being investigated is targeting human epidermal growth factor receptor 2 (HER2, ERBB2) considering that mutation or amplification of *ERBB2* gene has been identified in 9% of MIBC [45]. Trastuzumab deruxtecan is an ADC composed of trastuzumab, a monoclonal antibody targeting HER2 conjugated to deruxtecan, a derivative of the camptothecin analog exatecan, a DNA topoisomerase 1 inhibitor. This compound is being tested in combination with nivolumab in a phase I/II trial in patients with HER2 expression of IHC 1+, 2+ or 3+, progressed to prior platinum-based therapy (NCT03523572). RC48-ADC, an anti-HER2 monoclonal antibody, in under evaluation in two phase II trial in previously treated patients, one in HER2 negative (IHC 0 or 1+, NCT04073602) and one in HER2 overexpressed tumors (IHC 2+ or 3+, NCT03809013). PRS-343, a bivalent, bispecific fusion protein composed of an anti-HER2 monoclonal antibody linked to a CD137-targeting anticalin, is being investigated in HER2 positive solid tumor malignancy, including UC, for which standard therapies are not available. 

Ongoing phase I/II/III trials, either recruiting or active not recruiting, of target therapies in advanced or metastatic UC are summarized in Table 7, while miscellanea therapies are reported in Table 8.

## 4. Conclusions

In the recent years, the treatment scenario of metastatic UC has been enriched with several new therapeutic options. Immunotherapy is a very promising approach for this disease, but a high percentage of patients are still resistant to this type of treatment. In the future years, the results of the ongoing trials investigating ICIs in combination with target therapy or chemotherapy will assess if resistance to ICIs alone can be overcome. Promising treatment approaches are FGFR inhibitors and enfortumab vedotin. These two treatment strategies already showed good results in monotherapy and combination therapies with ICIs being tested. Other compounds, such as PARP inhibitors, mTOR inhibitors, anti-VEGF, tyrosine kinase inhibitors, HER2 targeting therapies, either alone or in several types of combinations, are being investigated in clinical trials.

The therapeutic approach to UC, which for many years has been dominated by platinum containing chemotherapy based on clinical and laboratory variable defining cisplatin eligibility, is now shifting toward a more personalized approach, based on the presence of molecular alteration (e.g., FGFR alterations) or PD-L1 expression.

## Figures and Tables

**Table 1:** Ongoing phase I/II/III trials of immune checkpoint inhibitors in combination with cytotoxic chemotherapy. ORR: overall response rate. PFS: progression-free survival. OS: overall survival.

NCT (Clinicaltrials.gov)	Phase	Setting	Cisplatin Fit/Unfit	Arm A	Arm B	Arm C	Compounds Description	Number of Patients	Primary Outcome	Status	Estimated Study Completion Date
**NCT03409458 (PAVE-1)**	Ib/IIa	First- or later-line	All	Avelumab + PT-112			PT-112: platinum- based agent belonging to the phosphaplatin family	52	Recommended dose of PT-112 to be used with avelumab	Recruiting	May 2020
**NCT02437370**	I	Second- or third-line	All	Pembrolizumab + docetaxel	Pembrolizumab + gemcitabine		Pembrolizumab: anti-PD-1	38	Safety	Recruiting	December 2020
**NCT03582475**	I	First- or later-line	All	Pembrolizumab + etoposide + cisplatin (or carboplatin)			Pembrolizumab: anti-PD-1	30	Durable response rate ORR Duration of response PFS OS Safety	Recruiting	September 2021
**NCT02853305 (KEYNOTE-361)**	III	First-line	All	Pembrolizumab	Pembrolizumab + gemcitabine + cisplatin (or carboplatin)	Placebo + gemcitabine + cisplatin (or carboplatin)	Pembrolizumab: anti-PD-1	990	PFS OS	Active, not recruiting	May 2020
**NCT02807636 (IMvigor130 trial)**	III	First-line	All	Atezolizumab	Atezolizumab + gemcitabine + cisplatin (or carboplatin)	Placebo + gemcitabine + cisplatin (or carboplatin)	Atezolizumab: anti-PD-L1	1200	PFS OS Safety	Active, not recruiting	November 2020
**NCT03093922**	II	First-line	All	Atezolizumab + gemcitabine + cisplatin	Atezolizumab + gemcitabine + cisplatin (modified schedule)	Atezolizumab + gemcitabine + cisplatin (modified schedule)	Atezolizumab: anti-PD-L1	74	ORR	Recruiting	March 2021
**NCT03967977**	III	First-line	All	Tislelizumab + gemcitabine + cisplatin (or carboplatin)	Placebo + gemcitabine + cisplatin (or carboplatin)		Tislelizumab: humanized monoclonal PD-1 antibody	420	OS	Recruiting	July 2022
**NCT03737123**	II	Second-line (no prior platinum chemotherapy)	Cisplatin ineligible	Atezolizumab + chemotherapy (docetaxel or gemcitabine + carboplatin)			Atezolizumab: anti-PD-L1	33	PFS	Recruiting	January 2022
**NCT03390595**	II	First-line	Cisplatin ineligible	Avelumab + gemcitabine + carboplatin	Gemcitabine + carboplatin		Avelumab: anti-PD-L1	85	ORR	Active, not recruiting	August 2020
**NCT03324282**	II	First-line	All	Avelumab + gemcitabine + cisplatin	Gemcitabine + cisplatin		Avelumab: anti-PD-L1	90	ORR Safety	Recruiting	December 2022
**NCT02581982**	II	Second- or later-line	All	Pembrolizumab + paclitaxel			Pembrolizumab: anti-PD-1	27	ORR	Recruiting	April 2020
**NCT03744793**	II	Second-line or third-line	All	Avelumab + pemetrexed			Avelumab: anti-PD-L1	25	ORR	Recruiting	January 2021
**NCT03575013**	I	Second- or later-line	All	Avelumab + paclitaxel			Avelumab: anti-PD-L1	21	Safety	Recruiting	May 2020

**Table 2:** Ongoing phase II/III trials of immune checkpoint inhibitor combined with other immune checkpoint inhibitors. ORR: overall response rate. PFS: progression-free survival. OS: overall survival.

NCT (Clinicaltrials.gov)	Phase	Setting	Cisplatin Fit/Unfit	Arm A	Arm B	Arm C	Compounds Description	Number of Patients	Primary Outcome	Status	Estimated Study Completion Date
**NCT03682068 (NILE)**	III	First-line	All	Durvalumab + gemcitabine + cisplatin (or carboplatin)	Durvalumab + tremelimumab + gemcitabine + cisplatin (or carboplatin)	Gemcitabine + cisplatin (or carboplatin)	Durvalumab: anti-PD-L1. Tremelimumab: anti-CTLA-4.	885	PFS OS	Recruiting	April 2022
**NCT03036098 (CheckMate-901)**	III	First-line	All	Nivolumab + ipilimumab	Gemcitabine + cisplatin (or carboplatin)	Nivolumab + gemcitabine + cisplatin (or carboplatin)	Nivolumab: anti-PD-1. Ipilimumab: anti-CTLA-4	990	OS in cisplatin ineligible OS in PD-L1 ≥ 1%	Recruiting	December 2022
**NCT03219775, (TITAN-TCC)**	II	First- or later-line	All	Nivolumab + ipilimumab			Nivolumab: anti-PD-1. Ipilimumab: anti-CTLA-4.	80	ORR	Recruiting	December 2020
**NCT02516241 (DANUBE)**	III	First-line	All	Durvalumab + tremelimumab	Durvalumab	Gemcitabine + cisplatin (or carboplatin)	Durvalumab: anti-PD-L1. Tremelimumab: anti-CTLA-4.	1126	OS	Active, not recruiting	May 2020
**NCT03430895**	II	First- or later-line	All	Durvalumab + tremelimuamb			Durvalumab: anti-PD-L1. Tremelimumab: anti-CTLA-4.	15	ORR	Active, not recruiting	January 2021

**Table 3:** Ongoing phase I/I/II trials of combinations between immune checkpoint inhibitors with antiangiogenic agents. ORR: overall response rate. PFS: progression-free survival. OS: overall survival.

NCT (Clinicaltrials.gov)	Phase	Setting	Cisplatin Fit/Unfit	Arm A	Arm B	Arm C	Compounds Description	Number of Patients	Primary Outcome	Status	Estimated Study Completion Date
**NCT02496208**	I	Second- or later-line	All	Nivolumab + cabozantinib	Nivolumab + ipilimumab + cabozantinib		Nivolumab: anti-PD-1. Ipilimumab: anti-CTLA-4. Cabozantinib: tyrosine kinase inhibitor	152	Safety	Recruiting	September 2020
**NCT02443324**	I	First- or later-line	All	Pembrolizumab + ramucirumab			Ramucirumab: anti-VEGF. Pembrolizumab: anti-PD-1.	155	Safety	Active, not recruiting	November 2020
**NCT03170960**	I/II	First- or later-line	All	Atezolizumab + cabozantinib			Atezolizumab: anti-PD-L1 Cabozantinib: tyrosine kinase inhibitor	1723	Safety ORR	Recruiting	December 2021
**NCT01552434**	I	Second- or later-line	All	Temsirolimus + bevacizumab + cetuximab	Temsirolimus + bevacizumab + valproic acid	Temsirolimus + bevacizumab	Temsirolimus: mTOR inhibitor. Bevacizumab: anti-VEGF. Cetuximab: anti-EGFR.	216	Safety	Recruiting	March 2021
**NCT03407976 (APPEASE)**	I/IIa	Second- or later-line	All	Pembrolizumab + apatinib			Apatinib: tyrosine kinase inhibitor. Pembrolizumab: anti-PD-1.	119	Safety ORR	Active, not recruiting	June 2023
**NCT03170960**	I/II	Second- or later-line (with prior ICI)	All	Expansion Cohort 2, 3, 4, 5: atezolizumab plus cabozantinib	Expansion cohort 19: cabozantinib		Atezolizumab: anti-PD-L1. Cabozantinib: tyrosine kinase inhibitor.	1732	Safety ORR	Recruiting	December 2021
**NCT03272217**	II	First-line	Cisplatin ineligible	Atezolizumab + bevacizumab			Bevacizumab: anti-VEGF	70	OS	Recruiting	June 2021
**NCT03472560 (JAVELIN Medley VEGF)**	II	Second- or later-line	Cisplatin ineligible	Avelumab + axitinib			Avelumab: anti-PD-L1. Axitinib: tyrosine kinase inhibitor.	61	OR	Active, not recruiting	September 2020
**NCT03898180 (LEAP-011 trial)**	III	First-line, PD-L1 ≥ 10%	Cisplatin ineligible	Pembrolizumab + lenvatinib	Pembrolizumab + placebo		Lenvatinib: tyrosine kinase inhibitor	694	PFS OS	Recruiting	December 2022
**NCT03534804 (PemCab)**	II	First-line	Cisplatin ineligible	Pembrolizumab + cabozantinib			Cabozantinib: tyrosine kinase inhibitor	39	ORR	Recruiting	September 2023
**NCT03824691 (ARCADIA)**	II	Second-line or third-line	All	Durvalumab + cabozantinib			Durvalumab: anti-PD-L1. Cabozantinib: tyrosine kinase inhibitor.	122	OS	Recruiting	February 2023
**NCT03866382**	II	First- or later-line	All	Nivolumab + ipilimumab + cabozantinib			Nivolumab: anti-PD-1. Ipilimumab: anti-CTLA-4. Cabozantinib: tyrosine kinase inhibitor.	186	ORR	Recruiting	February 2023
**NCT04066595 (CabUC)**	II	Second-line or third-line	All	Cabozantinib			Cabozantinib: tyrosine kinase inhibitor	88	ORR 6-month	Recruiting	September 2024
**NCT02717156**	II	Second- or later-line	All	Pembrolizumab + EphB4-HSA			EphB4-HSA: A recombinant fusion protein composed of the full-length extracellular domain (soluble) of human receptor tyrosine kinase ephrin type-B receptor 4 and fused to full-length human serum albumin, with potential anti-angiogenic and antineoplastic activities. Pembrolizumab: anti-PD-1.	60	Safety	Recruiting	November 2021
**NCT03606174**	II	First- or later-line	All	Nivolumab + sitravatinib			Sitravatinib: tyrosine kinase inhibitor able to inhibit VEGFR, PDGFR, KIT, RET and MET. Nivolumab: anti-PD-1.	330	ORR	Recruiting	September 2021

**Table 4:** Ongoing phase I/II/III trials of monotherapy immune checkpoint inhibitors. ORR: overall response rate. PFS: progression-free survival. OS: overall survival.

NCT (Clinicaltrials.gov)	Phase	Setting	Cisplatin Fit/Unfit	Arm A	Arm B	Arm C	Compounds Description	Number of Patients	Primary Outcome	Status	Estimated Study Completion Date
**NCT02500121**	II	Maintenance after SD, RP or RC to first-line chemotherapy	All	Pembrolizumab	Placebo		Pembrolizumab: anti-PD-1	108	6-month PFS	Active, not recruiting	January 2020
**NCT02603432 (JAVELIN Bladder 100)**	III	Maintenance after SD, RP or RC to first-line chemotherapy	All	Avelumab	Best supportive care		Avelumab: anti-PD-L1	700	OS	Active, not recruiting	June 2021
**NCT02928406 (SAUL)**	III	Second-, third or fourth- line	All	Atezolizumab			Atezolizumab: anti-PD-L1	1004	Safety	Active, not recruiting	March 2022
**NCT03113266**	II	Second- or later-line	All	Toripalimab (JS001)			Toripalimab: anti-PD-1	370	ORR	Recruiting	February 2022
**NCT03557918**	II	Second- or later-line	All	Tremelimumab			Tremelimumab: anti-CTLA-4	28	ORR	Recruiting	July 2021
**NCT03212404**	I	Second- or later-line (no prior ICI)	All	Cosibelimab (CK-301)			Cosibelimab: anti-PD-L1	500	Safety ORR	Recruiting	December 2021
**NCT03053466**	I	First- or later-line, PD-L1 ≥ 1%	All	APL-501			APL-501: anti-PD-1	114	Safety	Active, not recruiting	December 2021

**Table 5:** Ongoing phase I/II/III trials of novel immunotherapy approaches. ORR: overall response rate. PFS: progression-free survival. OS: overall survival.

NCT (Clinicaltrials.gov)	Phase	Setting	Cisplatin Fit/Unfit	Arm A	Arm B	Arm C	Compounds Description	Number of Patients	Primary Outcome	Status	Estimated Study Completion Date
**NCT03361865 (KEYNOTE-672/ECHO-307)**	III	First-line	Cisplatin ineligible	Pembrolizumab + epacadostat	Pembrolizumab + placebo		Epacadostat: IDO1 inhibitor. Pembrolizumab: anti-PD-1.	93	ORR	Active, not recruiting	September 2020
**NCT03374488**	III	Second- or later-line	All	Pembrolizumab + epacadostat	Pembrolizumab + placebo		Epacadostat: IDO1 inhibitor. Pembrolizumab: anti-PD-1.	84	ORR	Active, not recruiting	August 2020
**NCT02554812 (JAVELIN Medley)**	II	Second- or third-line	All	6 cohorts, different combinations with avelumab, PF-04518600 and utomilumab (PF-05082566)			PF-04518600: OX40 agonist. Utomilumab: 4-1BB agonist. Avelumab: anti-PD-L1.	620	Safety OR	Recruiting	December 2022
**NCT03785925 (PIVOT-10)**	II	First-line	Cisplatin ineligible	Nivolumab + Bempegaldesleukin (NKTR-214)			Bempegaldesleukin (NKTR-214): IL-2 pathway agonist designed to target CD122. Nivolumab: anti-PD-1.	205	ORR	Recruiting	March 2022
**NCT03513952**	II	Second- or later-line (prior platinum chemotherapy)	All	Atezolizumab + CYT-107	Atezolizumab		CYT-107: glycosylated recombinant human interleukin-7. Atezolizumab: anti-PD-L1.	54	ORR	Recruiting	December 2020
**NCT03915405**	I	Second- or later-line (no prior ICI, prior platinum chemotherapy)	All	Avelumab + KHK2455			KHK2455: IDO1 inhibitor. Avelumab: anti-PD-L1.	44	Safety	Recruiting	February 2022
**NCT03217747**	I/II	First- or later-line	All	6 cohorts, different combinations with utomilumab (PF-05082566), PF-04518600, and radiation therapy			PF-04518600: OX40 agonist Utomilumab: 4-1BB agonist	184	Safety CD8 immune markers	Recruiting	September 2023
**NCT04198766**	I	Second- or later-line	All	Pembrolizumab ± INBRX-106			INBRX-106: OX40 agonist. Pembrolizumab: anti-PD-1.	150	Safety	Recruiting	March 2023
**NCT02983045 (PIVOT-02)**	I/II	First- or later-line	All	Nivolumab + Bempegaldesleukin (NKTR-214)	Nivolumab + ipilimumab + NKTR-214		NKTR-214: IL-2 pathway agonist designed to target CD122. Nivolumab: anti-PD-1. Ipilimumab: anti-CTLA-4.	780	ORR	Recruiting	December 2021
**NCT03138889 (PROPEL)**	I/II	First- or later-line	All	Pembrolizumab + Bempegaldesleukin (NKTR-214)			Bempegaldesleukin (NKTR-214): an interleukin-2 pathway agonist that targets CD122. Pembrolizumab: anti-PD-1.	135	Safety ORR	Recruiting	June 2023
**NCT03809624**	I	Second- or later-line	All	INBRX-105			INBRX-105: next generation bispecific antibody targeting PD-L1 and 4-1BB, blocks inhibitory PD-1/PD-L1 axis and simultaneously activates essential co-stimulatory activity via 4-1BB	90	Safety	Recruiting	December 2021
**NCT03538028**	I	Second- or later-line	All	INCAGN02385			INCAGN02385: LAG-3 inhibitor	40	Safety	Recruiting	September 2020
**NCT03652077**	I	First- or later-line	All	INCAGN02390			INCAGN02390: TIM-3 inhibitor	41	Safety	Recruiting	January 2021
**NCT03126110**	I/II	First- or later-line	All	Nivolumab + INCAGN01876	Ipilimumab + INCAGN01876	Nivolumab + ipilimumab + INCAGN01876	INCAGN01876: GITR agonist. Nivolumab: anti-PD-1. Ipilimumab: anti-CTLA-4.	285	Safety ORR	Recruiting	October 2021
**NCT03185468**	I/II	First- or later-line	All	CAR-T				20	OS Safety	Recruiting	December 2020
**NCT03639714**	I/II	Second-line	All	Nivolumab + ipilimumab + GRT-C901 + GRT-R902			GRT-C901, GRT-R902: tumor vaccines. Nivolumab: anti-PD-1. Ipilimumab: anti-CTLA-4.	214	Safety ORR	Recruiting	March 2023
**NCT03228667 (QUILT-3.055)**	II	Second- or later-line	All	ALT-803 + ICI (nivolumab or pembrolizumab or avelumab or atezolizumab)			ALT-803: ALT-803: Superagonist Interleukin-15. Pembrolizumab: anti-PD-1. Nivolumab: anti-PD-1. Avelumab: anti-PD-L1. Atezolizumab: anti-PD-L1.	611	ORR	Recruiting	August 2020
**NCT03639714**	I	Second- or later-line	All	ARG1-18, 19, 20			ARG1-18, 19, 20: Arginase-1 Peptide Vaccine	10	Safety	Recruiting	June 2021
**NCT03917381**	I/II	Second- or later-line	All	GEN1046			GEN1046: bispecific antibody targeting PD-L1 and 4-1BB	192	Safety	Recruiting	February 2022
**NCT03517488 (DUET-2)**	I	Second- or later-line	All	XmAb20717			XmAb20717: A Fc-engineered bispecific antibody directed against the human negative immunoregulatory checkpoint receptors PD-1 and CTLA-4	154	Safety	Recruiting	March 2021
**NCT04044859**	I	Second- or later-line	All	Autologous genetically modified ADP-A2M4CD8 cells			Autologous genetically modified ADP-A2M4CD8 cells, directed to MAGE-A4, a member of the MAGE family expressed in a number of solid tumor types.	30	Safety	Recruiting	January 2021
**NCT03894618**	I	First-line (in platinum unfit) or second- or later-line	All	SL-279252			SL-279252: agonist redirected checkpoint fusion protein consisting of the extracellular domains of PD- 1 and OX40L, linked by a central Fc domain (PD1-Fc-OX40L).	87	Safety	Recruiting	April 2022
**NCT03849469 (DUET-4)**	I	Second- or later-line	All	XmAb^®^22841	Pembrolizumab + XmAb^®^22841		XmAb^®^22841: a Fc-engineered bispecific antibody directed against CTLA-4 and LAG-3. Pembrolizumab: anti-PD-1	242	Safety	Recruiting	March 2027
**NCT03752398 (DUET-3)**	I	Second- or later-line	All	XmAb^®^23104			XmAb23104: bispecific monoclonal antibody directed against PD-1 and inducible T-cell co-stimulator CD278	144	Safety	Recruiting	March 2025
**NCT03758781**	I	First- or later-line	All	IRX-2 Regimen plus Nivolumab			IRX-2 Regimen: cyclophosphamide and subcutaneous IRX-2, a cell-free mixture comprising a variety of naturally derived cytokines obtained from normal, unrelated donor lymphocytes with potential immunostimulatory activity. The cytokines in IRX-2 include interleukin-1, -2, -6, -8, -10, -12, tumor necrosis factor alpha, interferon-gamma and colony stimulating factors. Nivolumab: anti-PD-1.	100	Safety	Recruiting	February 2022
**NCT03841110**	I	First- or later-line	All	FT500	FT500 + nivolumab or pembrolizumab or atezolizumab		FT500: natural killer cell product that can bridge innate and adaptive immunity. Pembrolizumab: anti-PD-1. Nivolumab: anti-PD-1. Atezolizumab: anti-PD-L1.	76	Safety	Recruiting	June 2022
**NCT03329950**	I	Second- or later-line	All	CDX-1140	CDX-1140 + CDX-301	CDX-1140 + pembrolizumab	CDX-1140: anti-CD40, a key activator of immune response which is found on dendritic cells, macrophages and B cells and is also expressed on many cancer cells. CDX-301: recombinant human FMS-like tyrosine kinase-3 ligand that acts by uniquely binding FMS-like tyrosine kinase-3 (CD135). Pembrolizumab: anti-PD-1.	220	Safety	Recruiting	November 2021
**NCT03674567**	I/II	First-line (in platinum unfit) or second- or later-line	All	FLX475	FLX475 + pembrolizumab		FLX475: antagonist of C-C chemokine receptor type 4 with potential immunomodulatory and antineoplastic activities. FLX475 inhibits the binding of CCR4 to its signaling molecules, thereby blocking the recruitment of regulatory T cells to the tumor microenvironment. Pembrolizumab: anti-PD-1.	375	Safety ORR	Recruiting	August 2021
**NCT03970382**	I	Second- or later-line	All	NeoTCR-P1	NeoTCR-P1 + nivolumab		NeoTCR-P1: A preparation of autologous CD4- and CD8-positive T lymphocytes that have been engineered with site-specific nucleases to suppress the expression of most endogenous forms of the T-cell receptor and promote expression of a single, native T-cell receptor targeting a neoepitope presented on the surface of a patient’s tumor cells, with potential immunostimulating and antineoplastic activities. Nivolumab: anti-PD-1.	148	Safety	Recruiting	December 2023
**NCT03277352**	I/II	Second- or later-line	All	INCAGN01876 + Pembrolizumab + Epacadostat			Epacadostat: IDO1-inhibitor.INCAGN01876: anti-human glucocorticoid-induced tumor necrosis factor receptor agonistic humanized monoclonal antibody, with potential immune checkpoint modulating activity. Pembrolizumab: anti-PD-1.	10	Phase 1: safety. Phase 2: ORR and complete response rate	Active, not recruiting	May 2020
**NCT03250832 (CITRINO)**	I	Second- or later-line	All	TSR-033	TSR-033 + an anti-PD-1 agent		TSR-033: anti-LAG-3 monoclonal antibody	200	Safety	Recruiting	May 2021
**NCT03693612**	I/II	Second- or later-line	All	GSK3359609 plus tremelimumab			GSK3359609: agonistic antibody for the inducible T-cell co-stimulator (ICOS; CD278), with potential immune checkpoint inhibitory and antineoplastic activities. Tremelimumab: anti-CTLA-4.	114	Safety	Recruiting	April 2023
**NCT03739931**	I	First line in cisplatin ineligible and PD-L1 negative patients; second-line after platinum-containing chemotherapy	All	mRNA-2752	mRNA-2752 plus durvalumab		mRNA-2752: lipid nanoparticle encapsulating mRNAs encoding human OX40L, IL-23, and IL-36γ. Durvalumab: anti-PD-L1.	126	Safety	Recruiting	July 2021

**Table 6:** Ongoing phase I/II/III trials of PARP inhibitors. ORR: overall response rate. PFS: progression-free survival. OS: overall survival.

NCT (Clinicaltrials.gov)	Phase	Setting	Cisplatin Fit/Unfit	Arm A	Arm B	Arm C	Compounds Description	Number of Patients	Primary Outcome	Status	Estimated Study Completion Date
**NCT03682289**	II	Third- or later-line	All	AZD6738	AZD6738 plus olaparib		AZD6738: an orally available morpholino-pyrimidine-based inhibitor of ataxia telangiectasia and rad3 related kinase. Olaparib: PARP inhibitor.	68	ORR	Recruiting	March 2023
**NCT03945084**	II	Maintenance after first-line treatment	All	Niraparib + best supportive care	Best supportive care		Niraparib: PARP inhibitor	77	PFS	Recruiting	June 2024
**NCT03375307**	II	Second- or third-line	All	Olaparib			Olaparib: PARP inhibitor	150	ORR	Recruiting	August 2023
**NCT03448718**	II	Second- or later-lines; cisplatin ineligible; chemotherapy naïve	All	Olaparib			Olaparib: PARP inhibitor	30	ORR	Recruiting	March 2023
**NCT03459846** **(BAYOU)**	II	First-line	Cisplatin ineligible	Durvalumab + olaparib	Durvalumab + placebo		Durvalumab: anti-PD-L1. Olaparib: PARP inhibitor.	154	PFS	Active, not recruiting	September 2021
**NCT03992131**	I/II	Second- or later-line	All	Rucaparib and Lucitanib (phase Ib)	Rucaparib and Sacituzumab govitecan (phase Ib and II)		Sacituzumab govitecan: humanized anti-Trop-2 monoclonal antibody linked with SN-38, the active metabolite of irinotecan. Lucitanib: VEGFR 1, 2 and 3, FGFR 1 and 2, and PDGFR alpha and beta inhibitor. Rucaparib: PARP inhibitor.	329	Safety and tolerability, dose limiting toxicities (phase Ib), ORR (phase II)	Recruiting	March 2024
**NCT02546661 (BISCAY)**	I	Second- or third-line	All	8 cohorts: AZD4547; AZD4547 + durvalumab; durvalumab + olaparib; durvalumab + AZD1775; durvalumab; durvalumab + vistusertib; durvalumab + AZD9150; durvalumab + Selumetinib			AZD4547: FGFR-1, 2 and 3 inhibitor. AZD1775 (adavosertinib): inhibitor of the tyrosine kinase WEE1. Durvalumab: anti-PD-L1. Vistusertib: mTOR inhibitor. AZD9150 (danvatirsen): an antisense oligonucleotide targeting signal transducer and activator of transcription 3 (STAT3). Selumetinib: MEK or MAPK/ERK kinase 1 and 2 inhibitor.	156	Safety	Active, not recruiting	March 2020

**Table 7:** Ongoing phase I/II/III trials of target therapies. ORR: overall response rate. PFS: progression-free survival. OS: overall survival.

NCT (Clinicaltrials.gov)	Phase	Setting	Cisplatin Fit/Unfit	Arm A	Arm B	Arm C	Compounds Description	Number of Patients	Primary Outcome	Status	Estimated Study Completion Date
**NCT03980041 (MARIO-275)**	II	First- or later-line	All	Nivolumab + IPI-549	Nivolumab + placebo		IPI-549: PI3K inhibitor. Nivolumab: anti-PD-1.	160	ORR	Recruiting	November 2022
**NCT03745911**	II	Second- or later-line	All	Paclitaxel + TAK-228			TAK-228: PI3K/AKT/mTOR inhibitor	52	ORR	Recruiting	November 2020
**NCT00805129**	II	Second-, third or fourth- line	All	Everolimus			Everolimus: mTOR inhibitor	46	PFS Safety	Active, not recruiting	December 2020
**NCT02567409**	II	First-line or second-line (only with prior ICI)	All	Gemcitabine + cisplatin + berzosertib (M6620)	Gemcitabine + cisplatin		Berzosertib (M6620): ATR kinase inhibitor	90	PFS	Active, not recruiting	August 2020
**NCT03047213**	II	Second- or later-line	All	Sapanisertib			Sapanisertib: mTORC1 and mTORC2 inhibitor	209	ORR	Recruiting	June 2020
**NCT02535650**	II	Second- or later-line	All	Tipifarnib			Tipifarnib: farnesyltransferase inhibitor	18	6-month PFS	Recruiting	March 2020
**NCT01551030**	II	Second- or later-line	All	Buparlisib			Buparlisib: PI3K inhibitor	35	PFS	Active, not recruiting	March 2020
**NCT04073602**	II	Second- or later-line; HER-2 negative	All	RC48-ADC			RC48-ADC: anti-HER2 monoclonal antibody	20	ORR	Recruiting	October 2020
**NCT03809013**	II	Second- or later-line; HER2 overexpressed	All	RC48-ADC			RC48-ADC: anti-HER2 monoclonal antibody	60	ORR	Recruiting	December 2021
**NCT02795156**	II	Second-line	All	Afatinib or Regorafenib or Cabozantinib (based on specific genomic alterations on next-generation sequencing)			Afatinib, Regorafenib, Cabozantinib: tyrosine kinase inhibitor	160	ORR	Recruiting	December 2020
**NCT02872714 (FIGHT-201)**	II	Second- or later-line	All	Pemigatinib			Pemigatinib: FGFR inhibitor	240	ORR	Recruiting	August 2020
**NCT03410693**	II/III	Second- or later-line	All	Rogaratinib (BAY1163877)	Chemotherapy		Rogaratinib: FGFR inhibitor	171	OS	Active, not recruiting	November 2020
**(FORT-1)**											
**NCT04003610 (FIGHT-205)**	II	First-line	Cisplatin ineligible	Pemigatinib + Pembrolizumab	Pemigatinib	Chemotherapy or pembrolizumab	Pemigatinib: FGFR inhibitor. Pembrolizumab: anti-PD-1.	372	PFS	Recruiting	June 2024
**NCT03190174**	I/II	Second- or later-line	All	Nivolumab + ABI-009 (Nab-rapamycin)			ABI-009 (Nab-rapamycin): mTOR inhibitor. Nivolumab: anti-PD-1.	40	Safety	Recruiting	April 2021
**NCT03523572**	I/II	Second- or later-line	All	Trastuzumab Deruxtecan (DS-8201a) + Nivolumab			Trastuzumab Deruxtecan (DS-8201a): antibody drug conjugated composed of trastuzumab, a monoclonal antibody targeting HER2 conjugated to deruxtecan, a derivative of the camptothecin analog exatecan, a DNA topoisomerase 1 inhibitor. Nivolumab: anti-PD-1.	99	Safety, ORR	Recruiting	September 2020
**NCT03330561**	I	Third- or later-line	All	PRS-343			PRS-343: anti-HER2 monoclonal antibody linked to a CD137-targeting anticalin	78	Safety	Recruiting	September 2020
**NCT02546661 (BISCAY)**	I	Second- or third-line	All	8 cohorts: AZD4547; AZD4547 + durvalumab; durvalumab + olaparib; durvalumab + AZD1775; durvalumab;durvalumab + vistusertib; durvalumab + AZD9150; durvalumab + Selumetinib			AZD4547: FGFR-1, 2 and 3 inhibitor. AZD1775 (adavosertinib): inhibitor of the tyrosine kinase WEE1. Durvalumab: anti-PD-L1. Vistusertib: mTOR inhibitor. AZD9150 (danvatirsen): an antisense oligonucleotide targeting signal transducer and activator of transcription 3 (STAT3). Selumetinib: MEK or MAPK/ERK kinase 1 and 2 inhibitor.	156	Safety	Active, not recruiting	March 2020
**NCT02608125**	I	Third- or later-line	All	PRN1371			PRN1371: FGFR inhibitor	50	Safety	Recruiting	February 2021
**NCT03473756**	I/II	First-line	Cisplatin ineligible	Rogaratinib + Atezolizumab	Placebo + Atezolizumab		Rogaratinib: FGFR inhibitor	210	Safety, PFS	Recruiting	September 2024
**(FORT-2)**											
**NCT04045613**	I/II	First-line; later-lines after prior FGFR inhibitor	Cisplatin ineligible	Derazantinib	Derazantinib + Atezolizumab		Derazantinib: FGFR inhibitor. Atezolizumab: anti-PD-L1.	303	ORR; recommended phase 2 dose	Recruiting	May 2022
**NCT03473743**	I/II	Phase 1b: second- or later-line. Phase 2: first-line	Phase 2: cisplatin ineligible	Erdafitinib + Cetrelimab			Erdafitinib: FGFR inhibitor. Cetrelimab: anti-PD-1.	150	Safety, ORR	Recruiting	September 2021

**Table 8:** Ongoing phase I/II/III trials of miscellanea therapies. ORR: overall response rate. PFS: progression-free survival. OS: overall survival.

NCT (Clinicaltrials.gov)	Phase	Setting	Cisplatin Fit/Unfit	Arm A	Arm B	Arm C	Compounds Description	Number of Patients	Primary Outcome	Status	Estimated Study Completion Date
**NCT03854474**	I/II	Second- or later-line	All	Pembrolizumab + tazemetostat			Tazemetostat: Enhancer of zeste homolog 2 (EZH2) methyltransferase inhibitor. EZH2 is a histone-lysine N-methyltransferase enzyme participating in histone methylation and, ultimately, transcriptional repression. Pembrolizumab: anti-PD-1.	30	ORR	Recruiting	June 2020
**NCT04200963**	I	Second- or later-line	All	KYN-175			Aryl Hydrocarbon Receptor antagonist	53	Safety	Recruiting	September 2022
**NCT04007744**	I	First-line (in platinum unfit) or second- or later-line	All	Pembrolizumab + sonidegib			Sonidegib: Hedgehog signaling pathway inhibitor. Pembrolizumab: anti-PD-1.	45	Safety	Recruiting	June 2021
**NCT03829436**	I	First- or later-line	All	TPST-1120; TPST-1120 + nivolumab; TPST-1120 + docetaxel; TPST-1120 + cetuximab			TPST-1120: selective antagonist of peroxisome proliferator activated receptor alpha. Nivolumab: anti-PD-1. Cetuximab: anti-EGFR.	338	Safety	Recruiting	June 2024
**NCT02420847**	I/II	Second- or later-line	All	Ixazomib + Gemcitabine + Doxorubicin			Ixazomib: second generation proteasome inhibitor with potential antineoplastic activity	50	Safety	Active, not recruiting	July 2022
**NCT00365157**	I/II	Second- and third-line	All	Eribulin			Eribulin: microtubule-targeting agent	132	Maximum tolerated dose and recommended phase II dose, ORR	Active, not recruiting	December 2020
**NCT03179943**	II	First-line (in platinum unfit) or second- or later-line (with prior platinum-based chemotherapy or ICI)	All	Atezolizumab + guadecitabine			Guadecitabine: DNA methyltransferase (DNMT) inhibitor. Atezolizumab: anti- PD-L1.	53	Safety ORR	Recruiting	July 2022
**NCT03547973**	II	First-line (in platinum unfit) or second- or later-line (with prior platinum-based chemotherapy or ICI)	All	Sacituzumab govitecan (IMMU-132)			Sacituzumab govitecan (IMMU-132): Anti-Trop-2/SN-38 Antibody-Drug Conjugate	201	ORR	Recruiting	September 2021
**NCT04223856 (EV-302)**	III	First-line	Cisplatin or carboplatin eligible	Enfortumab vedotin + pembrolizumab	Enfortumab vedotin + pembrolizumab + cisplatin or carboplatin	Gemcitabine + cisplatin or carboplatin	Enfortumab vedotin: anti nectin- 4 monoclonal antibody liked to a micro- tubule-disrupting agent (monomethyl auristatin E). Pembrolizumab: anti-PD-1.	1095	PFS, OS	Recruiting	November 2023
**NCT03474107 (EV-301)**	III	Third- or later-line	All	Enfortumab vedotin	Chemotherapy (docetaxel, vinflunine, paclitaxel)		Enfortumab vedotin: anti nectin- 4 monoclonal antibody liked to a micro- tubule-disrupting agent (monomethyl auristatin E)	608	OS	Active, not recruiting	September 2021
